# Supplementary material for: Pyruvate Dehydrogenase Kinase 1 inhibition mediated oxidative phosphorylation enhancement in cartilage promotes osteoarthritis progression
Source: BMC Musculoskelet Disord. 2023 Jul 20;24:597. doi: 10.1186/s12891-023-06585-6 (PMC10357736; doi:10.1186/s12891-023-06585-6)
Supplement: Supplementary file 1 — Additional file 1. [file 12891_2023_6585_MOESM1_ESM.zip › KEGG.Gsea.1653622432464/KEGG_PATHWAYS_IN_CANCER.html]

Details for gene set KEGG\_PATHWAYS\_IN\_CANCER[GSEA]

|  || Dataset | 55457\_collapsed\_to\_symbols.cls.cls #OA\_versus\_normal.cls.cls #OA\_versus\_normal\_repos |
| Phenotype | cls.cls#OA\_versus\_normal\_repos |
| Upregulated in class | normal |
| GeneSet | KEGG\_PATHWAYS\_IN\_CANCER |
| Enrichment Score (ES) | -0.29421026 |
| Normalized Enrichment Score (NES) | -1.4878564 |
| Nominal p-value | 0.0013736264 |
| FDR q-value | 0.21318717 |
| FWER p-Value | 0.937 |
Table: GSEA Results Summary

  

Fig 1: Enrichment plot: KEGG\_PATHWAYS\_IN\_CANCER      
 Profile of the Running ES Score & Positions of GeneSet Members on the Rank Ordered List

  

| SYMBOL | TITLE | RANK IN GENE LIST | RANK METRIC SCORE | RUNNING ES | CORE ENRICHMENT || 1 | FZD1 | frizzled class receptor 1 [Source:HGNC Symbol;Acc:HGNC:4038] | 111 | 0.898 | 0.0003 | No |
| 2 | RALBP1 | ralA binding protein 1 [Source:HGNC Symbol;Acc:HGNC:9841] | 217 | 0.808 | 0.0001 | No |
| 3 | FZD7 | frizzled class receptor 7 [Source:HGNC Symbol;Acc:HGNC:4045] | 274 | 0.768 | 0.0034 | No |
| 4 | SMAD2 | SMAD family member 2 [Source:HGNC Symbol;Acc:HGNC:6768] | 275 | 0.768 | 0.0112 | No |
| 5 | WNT5B | Wnt family member 5B [Source:HGNC Symbol;Acc:HGNC:16265] | 327 | 0.734 | 0.0146 | No |
| 6 | RBX1 | ring-box 1 [Source:HGNC Symbol;Acc:HGNC:9928] | 348 | 0.724 | 0.0203 | No |
| 7 | FGF14 | fibroblast growth factor 14 [Source:HGNC Symbol;Acc:HGNC:3671] | 427 | 0.695 | 0.0212 | No |
| 8 | MLH1 | mutL homolog 1 [Source:HGNC Symbol;Acc:HGNC:7127] | 497 | 0.660 | 0.0224 | No |
| 9 | CSF1R | colony stimulating factor 1 receptor [Source:HGNC Symbol;Acc:HGNC:2433] | 713 | 0.590 | 0.0113 | No |
| 10 | MAPK9 | mitogen-activated protein kinase 9 [Source:HGNC Symbol;Acc:HGNC:6886] | 721 | 0.588 | 0.0167 | No |
| 11 | PLCG2 | phospholipase C gamma 2 [Source:HGNC Symbol;Acc:HGNC:9066] | 725 | 0.588 | 0.0224 | No |
| 12 | MSH2 | mutS homolog 2 [Source:HGNC Symbol;Acc:HGNC:7325] | 739 | 0.584 | 0.0273 | No |
| 13 | MAPK3 | mitogen-activated protein kinase 3 [Source:HGNC Symbol;Acc:HGNC:6877] | 757 | 0.579 | 0.0318 | No |
| 14 | CDKN2A | cyclin dependent kinase inhibitor 2A [Source:HGNC Symbol;Acc:HGNC:1787] | 799 | 0.570 | 0.0343 | No |
| 15 | FLT3LG | fms related receptor tyrosine kinase 3 ligand [Source:HGNC Symbol;Acc:HGNC:3766] | 894 | 0.544 | 0.0323 | No |
| 16 | FADD | Fas associated via death domain [Source:HGNC Symbol;Acc:HGNC:3573] | 924 | 0.534 | 0.0354 | No |
| 17 | CKS1B | CDC28 protein kinase regulatory subunit 1B [Source:HGNC Symbol;Acc:HGNC:19083] | 927 | 0.533 | 0.0407 | No |
| 18 | ELOB | elongin B [Source:HGNC Symbol;Acc:HGNC:11619] | 933 | 0.531 | 0.0457 | No |
| 19 | ERBB2 | erb-b2 receptor tyrosine kinase 2 [Source:HGNC Symbol;Acc:HGNC:3430] | 963 | 0.526 | 0.0487 | No |
| 20 | FGF18 | fibroblast growth factor 18 [Source:HGNC Symbol;Acc:HGNC:3674] | 969 | 0.525 | 0.0536 | No |
| 21 | FZD2 | frizzled class receptor 2 [Source:HGNC Symbol;Acc:HGNC:4040] | 1111 | 0.494 | 0.0474 | No |
| 22 | AKT1 | AKT serine/threonine kinase 1 [Source:HGNC Symbol;Acc:HGNC:391] | 1192 | 0.478 | 0.0459 | No |
| 23 | GSTP1 | glutathione S-transferase pi 1 [Source:HGNC Symbol;Acc:HGNC:4638] | 1260 | 0.465 | 0.0453 | No |
| 24 | CDK4 | cyclin dependent kinase 4 [Source:HGNC Symbol;Acc:HGNC:1773] | 1375 | 0.447 | 0.0407 | No |
| 25 | FGF23 | fibroblast growth factor 23 [Source:HGNC Symbol;Acc:HGNC:3680] | 1449 | 0.436 | 0.0394 | No |
| 26 | RAD51 | RAD51 recombinase [Source:HGNC Symbol;Acc:HGNC:9817] | 1581 | 0.416 | 0.0332 | No |
| 27 | CEBPA | CCAAT enhancer binding protein alpha [Source:HGNC Symbol;Acc:HGNC:1833] | 1750 | 0.392 | 0.0238 | No |
| 28 | FGF9 | fibroblast growth factor 9 [Source:HGNC Symbol;Acc:HGNC:3687] | 1833 | 0.380 | 0.0211 | No |
| 29 | DCC | DCC netrin 1 receptor [Source:HGNC Symbol;Acc:HGNC:2701] | 1862 | 0.378 | 0.0227 | No |
| 30 | WNT5A | Wnt family member 5A [Source:HGNC Symbol;Acc:HGNC:12784] | 1995 | 0.361 | 0.0159 | No |
| 31 | WNT3 | Wnt family member 3 [Source:HGNC Symbol;Acc:HGNC:12782] | 2003 | 0.360 | 0.0190 | No |
| 32 | CRK | "CRK proto-oncogene, adaptor protein [Source:HGNC Symbol;Acc:HGNC:2362]" | 2018 | 0.360 | 0.0215 | No |
| 33 | CTNNA3 | catenin alpha 3 [Source:HGNC Symbol;Acc:HGNC:2511] | 2030 | 0.358 | 0.0243 | No |
| 34 | KIT | "KIT proto-oncogene, receptor tyrosine kinase [Source:HGNC Symbol;Acc:HGNC:6342]" | 2117 | 0.348 | 0.0210 | No |
| 35 | LAMC3 | laminin subunit gamma 3 [Source:HGNC Symbol;Acc:HGNC:6494] | 2177 | 0.341 | 0.0197 | No |
| 36 | PIK3CB | "phosphatidylinositol-4,5-bisphosphate 3-kinase catalytic subunit beta [Source:HGNC Symbol;Acc:HGNC:8976]" | 2186 | 0.340 | 0.0225 | No |
| 37 | VEGFC | vascular endothelial growth factor C [Source:HGNC Symbol;Acc:HGNC:12682] | 2239 | 0.335 | 0.0218 | No |
| 38 | CRKL | "CRK like proto-oncogene, adaptor protein [Source:HGNC Symbol;Acc:HGNC:2363]" | 2283 | 0.329 | 0.0217 | No |
| 39 | IGF1 | insulin like growth factor 1 [Source:HGNC Symbol;Acc:HGNC:5464] | 2286 | 0.328 | 0.0249 | No |
| 40 | EGF | epidermal growth factor [Source:HGNC Symbol;Acc:HGNC:3229] | 2399 | 0.315 | 0.0191 | No |
| 41 | CCNE2 | cyclin E2 [Source:HGNC Symbol;Acc:HGNC:1590] | 2469 | 0.306 | 0.0168 | No |
| 42 | TGFB1 | transforming growth factor beta 1 [Source:HGNC Symbol;Acc:HGNC:11766] | 2490 | 0.303 | 0.0182 | No |
| 43 | HGF | hepatocyte growth factor [Source:HGNC Symbol;Acc:HGNC:4893] | 2547 | 0.297 | 0.0168 | No |
| 44 | CDH1 | cadherin 1 [Source:HGNC Symbol;Acc:HGNC:1748] | 2589 | 0.292 | 0.0165 | No |
| 45 | KLK3 | kallikrein related peptidase 3 [Source:HGNC Symbol;Acc:HGNC:6364] | 2627 | 0.287 | 0.0165 | No |
| 46 | DVL2 | dishevelled segment polarity protein 2 [Source:HGNC Symbol;Acc:HGNC:3086] | 2693 | 0.280 | 0.0141 | No |
| 47 | WNT2 | Wnt family member 2 [Source:HGNC Symbol;Acc:HGNC:12780] | 2700 | 0.279 | 0.0165 | No |
| 48 | FGFR3 | fibroblast growth factor receptor 3 [Source:HGNC Symbol;Acc:HGNC:3690] | 2812 | 0.269 | 0.0104 | No |
| 49 | FGF20 | fibroblast growth factor 20 [Source:HGNC Symbol;Acc:HGNC:3677] | 2849 | 0.265 | 0.0102 | No |
| 50 | GLI3 | GLI family zinc finger 3 [Source:HGNC Symbol;Acc:HGNC:4319] | 2861 | 0.264 | 0.0120 | No |
| 51 | PDGFRA | platelet derived growth factor receptor alpha [Source:HGNC Symbol;Acc:HGNC:8803] | 2943 | 0.256 | 0.0082 | No |
| 52 | CDKN1B | cyclin dependent kinase inhibitor 1B [Source:HGNC Symbol;Acc:HGNC:1785] | 2984 | 0.251 | 0.0076 | No |
| 53 | NTRK1 | neurotrophic receptor tyrosine kinase 1 [Source:HGNC Symbol;Acc:HGNC:8031] | 3015 | 0.248 | 0.0077 | No |
| 54 | CASP3 | caspase 3 [Source:HGNC Symbol;Acc:HGNC:1504] | 3202 | 0.230 | -0.0048 | No |
| 55 | RALB | RAS like proto-oncogene B [Source:HGNC Symbol;Acc:HGNC:9840] | 3224 | 0.228 | -0.0041 | No |
| 56 | TPM3 | tropomyosin 3 [Source:HGNC Symbol;Acc:HGNC:12012] | 3484 | 0.204 | -0.0226 | No |
| 57 | ARNT2 | aryl hydrocarbon receptor nuclear translocator 2 [Source:HGNC Symbol;Acc:HGNC:16876] | 3512 | 0.201 | -0.0227 | No |
| 58 | APPL1 | "adaptor protein, phosphotyrosine interacting with PH domain and leucine zipper 1 [Source:HGNC Symbol;Acc:HGNC:24035]" | 3532 | 0.199 | -0.0222 | No |
| 59 | COL4A4 | collagen type IV alpha 4 chain [Source:HGNC Symbol;Acc:HGNC:2206] | 3592 | 0.195 | -0.0249 | No |
| 60 | MMP1 | matrix metallopeptidase 1 [Source:HGNC Symbol;Acc:HGNC:7155] | 3625 | 0.191 | -0.0255 | No |
| 61 | CUL2 | cullin 2 [Source:HGNC Symbol;Acc:HGNC:2552] | 3660 | 0.189 | -0.0263 | No |
| 62 | LAMB3 | laminin subunit beta 3 [Source:HGNC Symbol;Acc:HGNC:6490] | 3679 | 0.188 | -0.0259 | No |
| 63 | PAX8 | paired box 8 [Source:HGNC Symbol;Acc:HGNC:8622] | 3680 | 0.187 | -0.0240 | No |
| 64 | TFG | trafficking from ER to golgi regulator [Source:HGNC Symbol;Acc:HGNC:11758] | 3719 | 0.183 | -0.0251 | No |
| 65 | PRKCA | protein kinase C alpha [Source:HGNC Symbol;Acc:HGNC:9393] | 3728 | 0.183 | -0.0239 | No |
| 66 | BAD | BCL2 associated agonist of cell death [Source:HGNC Symbol;Acc:HGNC:936] | 3788 | 0.178 | -0.0268 | No |
| 67 | CSF2RA | colony stimulating factor 2 receptor subunit alpha [Source:HGNC Symbol;Acc:HGNC:2435] | 3797 | 0.178 | -0.0256 | No |
| 68 | MAX | MYC associated factor X [Source:HGNC Symbol;Acc:HGNC:6913] | 3804 | 0.176 | -0.0243 | No |
| 69 | FAS | Fas cell surface death receptor [Source:HGNC Symbol;Acc:HGNC:11920] | 3828 | 0.173 | -0.0244 | No |
| 70 | RAC3 | Rac family small GTPase 3 [Source:HGNC Symbol;Acc:HGNC:9803] | 3849 | 0.171 | -0.0243 | No |
| 71 | BRCA2 | BRCA2 DNA repair associated [Source:HGNC Symbol;Acc:HGNC:1101] | 3853 | 0.170 | -0.0228 | No |
| 72 | FASLG | Fas ligand [Source:HGNC Symbol;Acc:HGNC:11936] | 3931 | 0.162 | -0.0273 | No |
| 73 | RELA | "RELA proto-oncogene, NF-kB subunit [Source:HGNC Symbol;Acc:HGNC:9955]" | 3942 | 0.161 | -0.0264 | No |
| 74 | CTNNA1 | catenin alpha 1 [Source:HGNC Symbol;Acc:HGNC:2509] | 4003 | 0.156 | -0.0296 | No |
| 75 | SMAD3 | SMAD family member 3 [Source:HGNC Symbol;Acc:HGNC:6769] | 4061 | 0.150 | -0.0326 | No |
| 76 | VHL | von Hippel-Lindau tumor suppressor [Source:HGNC Symbol;Acc:HGNC:12687] | 4142 | 0.143 | -0.0375 | No |
| 77 | RARB | retinoic acid receptor beta [Source:HGNC Symbol;Acc:HGNC:9865] | 4166 | 0.141 | -0.0379 | No |
| 78 | WNT2B | Wnt family member 2B [Source:HGNC Symbol;Acc:HGNC:12781] | 4194 | 0.138 | -0.0387 | No |
| 79 | FGF1 | fibroblast growth factor 1 [Source:HGNC Symbol;Acc:HGNC:3665] | 4436 | 0.118 | -0.0566 | No |
| 80 | STAT1 | signal transducer and activator of transcription 1 [Source:HGNC Symbol;Acc:HGNC:11362] | 4495 | 0.113 | -0.0601 | No |
| 81 | DVL1 | dishevelled segment polarity protein 1 [Source:HGNC Symbol;Acc:HGNC:3084] | 4612 | 0.104 | -0.0682 | No |
| 82 | NFKB1 | nuclear factor kappa B subunit 1 [Source:HGNC Symbol;Acc:HGNC:7794] | 4626 | 0.102 | -0.0682 | No |
| 83 | FLT3 | fms related receptor tyrosine kinase 3 [Source:HGNC Symbol;Acc:HGNC:3765] | 4657 | 0.099 | -0.0696 | No |
| 84 | WNT11 | Wnt family member 11 [Source:HGNC Symbol;Acc:HGNC:12776] | 4688 | 0.097 | -0.0710 | No |
| 85 | MITF | melanocyte inducing transcription factor [Source:HGNC Symbol;Acc:HGNC:7105] | 4759 | 0.090 | -0.0757 | No |
| 86 | FGF8 | fibroblast growth factor 8 [Source:HGNC Symbol;Acc:HGNC:3686] | 4761 | 0.090 | -0.0748 | No |
| 87 | FGF5 | fibroblast growth factor 5 [Source:HGNC Symbol;Acc:HGNC:3683] | 4990 | 0.074 | -0.0922 | No |
| 88 | FGF16 | fibroblast growth factor 16 [Source:HGNC Symbol;Acc:HGNC:3672] | 5013 | 0.072 | -0.0932 | No |
| 89 | TCF7L2 | transcription factor 7 like 2 [Source:HGNC Symbol;Acc:HGNC:11641] | 5034 | 0.070 | -0.0941 | No |
| 90 | BIRC2 | baculoviral IAP repeat containing 2 [Source:HGNC Symbol;Acc:HGNC:590] | 5054 | 0.069 | -0.0949 | No |
| 91 | CTBP2 | C-terminal binding protein 2 [Source:HGNC Symbol;Acc:HGNC:2495] | 5066 | 0.067 | -0.0951 | No |
| 92 | FGF13 | fibroblast growth factor 13 [Source:HGNC Symbol;Acc:HGNC:3670] | 5070 | 0.067 | -0.0947 | No |
| 93 | LAMB4 | laminin subunit beta 4 [Source:HGNC Symbol;Acc:HGNC:6491] | 5111 | 0.063 | -0.0972 | No |
| 94 | E2F1 | E2F transcription factor 1 [Source:HGNC Symbol;Acc:HGNC:3113] | 5137 | 0.061 | -0.0986 | No |
| 95 | PIAS3 | protein inhibitor of activated STAT 3 [Source:HGNC Symbol;Acc:HGNC:16861] | 5195 | 0.057 | -0.1025 | No |
| 96 | TGFBR1 | transforming growth factor beta receptor 1 [Source:HGNC Symbol;Acc:HGNC:11772] | 5280 | 0.050 | -0.1087 | No |
| 97 | FGF7 | fibroblast growth factor 7 [Source:HGNC Symbol;Acc:HGNC:3685] | 5386 | 0.041 | -0.1166 | No |
| 98 | FZD5 | frizzled class receptor 5 [Source:HGNC Symbol;Acc:HGNC:4043] | 5392 | 0.041 | -0.1166 | No |
| 99 | LAMA4 | laminin subunit alpha 4 [Source:HGNC Symbol;Acc:HGNC:6484] | 5473 | 0.035 | -0.1226 | No |
| 100 | RUNX1T1 | RUNX1 partner transcriptional co-repressor 1 [Source:HGNC Symbol;Acc:HGNC:1535] | 5503 | 0.032 | -0.1246 | No |
| 101 | TP53 | tumor protein p53 [Source:HGNC Symbol;Acc:HGNC:11998] | 5511 | 0.032 | -0.1248 | No |
| 102 | STAT5B | signal transducer and activator of transcription 5B [Source:HGNC Symbol;Acc:HGNC:11367] | 5554 | 0.028 | -0.1279 | No |
| 103 | BCL2 | BCL2 apoptosis regulator [Source:HGNC Symbol;Acc:HGNC:990] | 5609 | 0.023 | -0.1319 | No |
| 104 | MECOM | MDS1 and EVI1 complex locus [Source:HGNC Symbol;Acc:HGNC:3498] | 5622 | 0.022 | -0.1326 | No |
| 105 | CCNE1 | cyclin E1 [Source:HGNC Symbol;Acc:HGNC:1589] | 5663 | 0.018 | -0.1356 | No |
| 106 | GRB2 | growth factor receptor bound protein 2 [Source:HGNC Symbol;Acc:HGNC:4566] | 5697 | 0.016 | -0.1381 | No |
| 107 | BID | BH3 interacting domain death agonist [Source:HGNC Symbol;Acc:HGNC:1050] | 5715 | 0.014 | -0.1393 | No |
| 108 | FH | fumarate hydratase [Source:HGNC Symbol;Acc:HGNC:3700] | 5729 | 0.013 | -0.1402 | No |
| 109 | TRAF6 | TNF receptor associated factor 6 [Source:HGNC Symbol;Acc:HGNC:12036] | 5761 | 0.011 | -0.1425 | No |
| 110 | WNT6 | Wnt family member 6 [Source:HGNC Symbol;Acc:HGNC:12785] | 5781 | 0.010 | -0.1440 | No |
| 111 | IKBKB | inhibitor of nuclear factor kappa B kinase subunit beta [Source:HGNC Symbol;Acc:HGNC:5960] | 5914 | 0.001 | -0.1544 | No |
| 112 | TPR | "translocated promoter region, nuclear basket protein [Source:HGNC Symbol;Acc:HGNC:12017]" | 5919 | 0.001 | -0.1547 | No |
| 113 | BIRC3 | baculoviral IAP repeat containing 3 [Source:HGNC Symbol;Acc:HGNC:591] | 5942 | -0.001 | -0.1565 | No |
| 114 | COL4A6 | collagen type IV alpha 6 chain [Source:HGNC Symbol;Acc:HGNC:2208] | 5975 | -0.003 | -0.1590 | No |
| 115 | PPARG | peroxisome proliferator activated receptor gamma [Source:HGNC Symbol;Acc:HGNC:9236] | 6006 | -0.005 | -0.1613 | No |
| 116 | RB1 | RB transcriptional corepressor 1 [Source:HGNC Symbol;Acc:HGNC:9884] | 6013 | -0.006 | -0.1617 | No |
| 117 | FZD8 | frizzled class receptor 8 [Source:HGNC Symbol;Acc:HGNC:4046] | 6084 | -0.011 | -0.1672 | No |
| 118 | RAC2 | Rac family small GTPase 2 [Source:HGNC Symbol;Acc:HGNC:9802] | 6098 | -0.013 | -0.1681 | No |
| 119 | TRAF3 | TNF receptor associated factor 3 [Source:HGNC Symbol;Acc:HGNC:12033] | 6108 | -0.013 | -0.1687 | No |
| 120 | RAF1 | "Raf-1 proto-oncogene, serine/threonine kinase [Source:HGNC Symbol;Acc:HGNC:9829]" | 6187 | -0.019 | -0.1747 | No |
| 121 | WNT7B | Wnt family member 7B [Source:HGNC Symbol;Acc:HGNC:12787] | 6230 | -0.023 | -0.1778 | No |
| 122 | TGFB3 | transforming growth factor beta 3 [Source:HGNC Symbol;Acc:HGNC:11769] | 6293 | -0.027 | -0.1824 | No |
| 123 | EGLN1 | egl-9 family hypoxia inducible factor 1 [Source:HGNC Symbol;Acc:HGNC:1232] | 6364 | -0.032 | -0.1877 | No |
| 124 | PML | PML nuclear body scaffold [Source:HGNC Symbol;Acc:HGNC:9113] | 6421 | -0.037 | -0.1917 | No |
| 125 | PRKCB | protein kinase C beta [Source:HGNC Symbol;Acc:HGNC:9395] | 6453 | -0.039 | -0.1938 | No |
| 126 | FOXO1 | forkhead box O1 [Source:HGNC Symbol;Acc:HGNC:3819] | 6511 | -0.043 | -0.1979 | No |
| 127 | ELOC | elongin C [Source:HGNC Symbol;Acc:HGNC:11617] | 6556 | -0.046 | -0.2009 | No |
| 128 | BCL2L1 | BCL2 like 1 [Source:HGNC Symbol;Acc:HGNC:992] | 6698 | -0.055 | -0.2116 | No |
| 129 | FZD9 | frizzled class receptor 9 [Source:HGNC Symbol;Acc:HGNC:4047] | 6812 | -0.064 | -0.2199 | No |
| 130 | MAPK1 | mitogen-activated protein kinase 1 [Source:HGNC Symbol;Acc:HGNC:6871] | 6853 | -0.068 | -0.2224 | No |
| 131 | PDGFRB | platelet derived growth factor receptor beta [Source:HGNC Symbol;Acc:HGNC:8804] | 6914 | -0.073 | -0.2264 | No |
| 132 | RXRG | retinoid X receptor gamma [Source:HGNC Symbol;Acc:HGNC:10479] | 6947 | -0.075 | -0.2282 | No |
| 133 | TGFB2 | transforming growth factor beta 2 [Source:HGNC Symbol;Acc:HGNC:11768] | 6985 | -0.079 | -0.2303 | No |
| 134 | STAT5A | signal transducer and activator of transcription 5A [Source:HGNC Symbol;Acc:HGNC:11366] | 6988 | -0.079 | -0.2297 | No |
| 135 | JAK1 | Janus kinase 1 [Source:HGNC Symbol;Acc:HGNC:6190] | 6995 | -0.079 | -0.2294 | No |
| 136 | MMP2 | matrix metallopeptidase 2 [Source:HGNC Symbol;Acc:HGNC:7166] | 7037 | -0.082 | -0.2318 | No |
| 137 | ITGAV | integrin subunit alpha V [Source:HGNC Symbol;Acc:HGNC:6150] | 7067 | -0.085 | -0.2332 | No |
| 138 | SLC2A1 | solute carrier family 2 member 1 [Source:HGNC Symbol;Acc:HGNC:11005] | 7089 | -0.087 | -0.2340 | No |
| 139 | WNT7A | Wnt family member 7A [Source:HGNC Symbol;Acc:HGNC:12786] | 7166 | -0.093 | -0.2391 | No |
| 140 | FGF2 | fibroblast growth factor 2 [Source:HGNC Symbol;Acc:HGNC:3676] | 7183 | -0.095 | -0.2394 | No |
| 141 | RAC1 | Rac family small GTPase 1 [Source:HGNC Symbol;Acc:HGNC:9801] | 7192 | -0.096 | -0.2391 | No |
| 142 | APC2 | APC regulator of WNT signaling pathway 2 [Source:HGNC Symbol;Acc:HGNC:24036] | 7271 | -0.101 | -0.2443 | No |
| 143 | MDM2 | MDM2 proto-oncogene [Source:HGNC Symbol;Acc:HGNC:6973] | 7273 | -0.101 | -0.2433 | No |
| 144 | NRAS | "NRAS proto-oncogene, GTPase [Source:HGNC Symbol;Acc:HGNC:7989]" | 7300 | -0.102 | -0.2444 | No |
| 145 | PTK2 | protein tyrosine kinase 2 [Source:HGNC Symbol;Acc:HGNC:9611] | 7315 | -0.103 | -0.2444 | No |
| 146 | MET | "MET proto-oncogene, receptor tyrosine kinase [Source:HGNC Symbol;Acc:HGNC:7029]" | 7322 | -0.104 | -0.2439 | No |
| 147 | FGFR2 | fibroblast growth factor receptor 2 [Source:HGNC Symbol;Acc:HGNC:3689] | 7374 | -0.108 | -0.2468 | No |
| 148 | RXRA | retinoid X receptor alpha [Source:HGNC Symbol;Acc:HGNC:10477] | 7381 | -0.109 | -0.2462 | No |
| 149 | TCF7L1 | transcription factor 7 like 1 [Source:HGNC Symbol;Acc:HGNC:11640] | 7383 | -0.109 | -0.2452 | No |
| 150 | LAMA2 | laminin subunit alpha 2 [Source:HGNC Symbol;Acc:HGNC:6482] | 7392 | -0.110 | -0.2447 | No |
| 151 | PIK3CD | "phosphatidylinositol-4,5-bisphosphate 3-kinase catalytic subunit delta [Source:HGNC Symbol;Acc:HGNC:8977]" | 7405 | -0.111 | -0.2445 | No |
| 152 | LEF1 | lymphoid enhancer binding factor 1 [Source:HGNC Symbol;Acc:HGNC:6551] | 7467 | -0.116 | -0.2482 | No |
| 153 | TRAF1 | TNF receptor associated factor 1 [Source:HGNC Symbol;Acc:HGNC:12031] | 7474 | -0.116 | -0.2475 | No |
| 154 | CCND1 | cyclin D1 [Source:HGNC Symbol;Acc:HGNC:1582] | 7512 | -0.119 | -0.2492 | No |
| 155 | FGF3 | fibroblast growth factor 3 [Source:HGNC Symbol;Acc:HGNC:3681] | 7637 | -0.130 | -0.2578 | No |
| 156 | WNT10B | Wnt family member 10B [Source:HGNC Symbol;Acc:HGNC:12775] | 7700 | -0.134 | -0.2613 | No |
| 157 | PIK3CA | "phosphatidylinositol-4,5-bisphosphate 3-kinase catalytic subunit alpha [Source:HGNC Symbol;Acc:HGNC:8975]" | 7733 | -0.136 | -0.2625 | No |
| 158 | BMP4 | bone morphogenetic protein 4 [Source:HGNC Symbol;Acc:HGNC:1071] | 7816 | -0.142 | -0.2676 | No |
| 159 | LAMB2 | laminin subunit beta 2 [Source:HGNC Symbol;Acc:HGNC:6487] | 7841 | -0.144 | -0.2680 | No |
| 160 | ITGA2 | integrin subunit alpha 2 [Source:HGNC Symbol;Acc:HGNC:6137] | 7857 | -0.145 | -0.2677 | No |
| 161 | FGFR1 | fibroblast growth factor receptor 1 [Source:HGNC Symbol;Acc:HGNC:3688] | 7905 | -0.148 | -0.2700 | No |
| 162 | HDAC1 | histone deacetylase 1 [Source:HGNC Symbol;Acc:HGNC:4852] | 7911 | -0.148 | -0.2689 | No |
| 163 | PIK3CG | "phosphatidylinositol-4,5-bisphosphate 3-kinase catalytic subunit gamma [Source:HGNC Symbol;Acc:HGNC:8978]" | 7953 | -0.152 | -0.2706 | No |
| 164 | HRAS | "HRas proto-oncogene, GTPase [Source:HGNC Symbol;Acc:HGNC:5173]" | 7977 | -0.153 | -0.2709 | No |
| 165 | AKT3 | AKT serine/threonine kinase 3 [Source:HGNC Symbol;Acc:HGNC:393] | 8008 | -0.156 | -0.2717 | No |
| 166 | SOS2 | SOS Ras/Rho guanine nucleotide exchange factor 2 [Source:HGNC Symbol;Acc:HGNC:11188] | 8058 | -0.160 | -0.2739 | No |
| 167 | E2F2 | E2F transcription factor 2 [Source:HGNC Symbol;Acc:HGNC:3114] | 8090 | -0.162 | -0.2748 | No |
| 168 | ARAF | "A-Raf proto-oncogene, serine/threonine kinase [Source:HGNC Symbol;Acc:HGNC:646]" | 8092 | -0.163 | -0.2732 | No |
| 169 | CASP9 | caspase 9 [Source:HGNC Symbol;Acc:HGNC:1511] | 8142 | -0.166 | -0.2754 | No |
| 170 | FZD4 | frizzled class receptor 4 [Source:HGNC Symbol;Acc:HGNC:4042] | 8159 | -0.168 | -0.2750 | No |
| 171 | BRAF | "B-Raf proto-oncogene, serine/threonine kinase [Source:HGNC Symbol;Acc:HGNC:1097]" | 8171 | -0.169 | -0.2741 | No |
| 172 | AR | androgen receptor [Source:HGNC Symbol;Acc:HGNC:644] | 8217 | -0.173 | -0.2760 | No |
| 173 | RASSF1 | Ras association domain family member 1 [Source:HGNC Symbol;Acc:HGNC:9882] | 8229 | -0.173 | -0.2751 | No |
| 174 | TGFBR2 | transforming growth factor beta receptor 2 [Source:HGNC Symbol;Acc:HGNC:11773] | 8241 | -0.174 | -0.2742 | No |
| 175 | TRAF2 | TNF receptor associated factor 2 [Source:HGNC Symbol;Acc:HGNC:12032] | 8322 | -0.180 | -0.2787 | No |
| 176 | FZD10 | frizzled class receptor 10 [Source:HGNC Symbol;Acc:HGNC:4039] | 8354 | -0.182 | -0.2794 | No |
| 177 | FZD6 | frizzled class receptor 6 [Source:HGNC Symbol;Acc:HGNC:4044] | 8379 | -0.184 | -0.2794 | No |
| 178 | CDK6 | cyclin dependent kinase 6 [Source:HGNC Symbol;Acc:HGNC:1777] | 8393 | -0.185 | -0.2786 | No |
| 179 | CYCS | "cytochrome c, somatic [Source:HGNC Symbol;Acc:HGNC:19986]" | 8410 | -0.186 | -0.2780 | No |
| 180 | STAT3 | signal transducer and activator of transcription 3 [Source:HGNC Symbol;Acc:HGNC:11364] | 8453 | -0.190 | -0.2794 | No |
| 181 | PRKCG | protein kinase C gamma [Source:HGNC Symbol;Acc:HGNC:9402] | 8582 | -0.200 | -0.2875 | No |
| 182 | CCNA1 | cyclin A1 [Source:HGNC Symbol;Acc:HGNC:1577] | 8584 | -0.200 | -0.2856 | No |
| 183 | DAPK1 | death associated protein kinase 1 [Source:HGNC Symbol;Acc:HGNC:2674] | 8588 | -0.200 | -0.2838 | No |
| 184 | RHOA | ras homolog family member A [Source:HGNC Symbol;Acc:HGNC:667] | 8601 | -0.201 | -0.2827 | No |
| 185 | SKP2 | S-phase kinase associated protein 2 [Source:HGNC Symbol;Acc:HGNC:10901] | 8625 | -0.203 | -0.2825 | No |
| 186 | VEGFB | vascular endothelial growth factor B [Source:HGNC Symbol;Acc:HGNC:12681] | 8673 | -0.206 | -0.2841 | No |
| 187 | MMP9 | matrix metallopeptidase 9 [Source:HGNC Symbol;Acc:HGNC:7176] | 8698 | -0.209 | -0.2839 | No |
| 188 | TGFA | transforming growth factor alpha [Source:HGNC Symbol;Acc:HGNC:11765] | 8748 | -0.212 | -0.2856 | No |
| 189 | XIAP | X-linked inhibitor of apoptosis [Source:HGNC Symbol;Acc:HGNC:592] | 8750 | -0.212 | -0.2836 | No |
| 190 | DVL3 | dishevelled segment polarity protein 3 [Source:HGNC Symbol;Acc:HGNC:3087] | 8756 | -0.213 | -0.2818 | No |
| 191 | PIK3R3 | phosphoinositide-3-kinase regulatory subunit 3 [Source:HGNC Symbol;Acc:HGNC:8981] | 8801 | -0.217 | -0.2831 | No |
| 192 | PIK3R5 | phosphoinositide-3-kinase regulatory subunit 5 [Source:HGNC Symbol;Acc:HGNC:30035] | 8804 | -0.217 | -0.2811 | No |
| 193 | WNT16 | Wnt family member 16 [Source:HGNC Symbol;Acc:HGNC:16267] | 8808 | -0.218 | -0.2791 | No |
| 194 | DAPK2 | death associated protein kinase 2 [Source:HGNC Symbol;Acc:HGNC:2675] | 8818 | -0.219 | -0.2776 | No |
| 195 | PTCH1 | patched 1 [Source:HGNC Symbol;Acc:HGNC:9585] | 8837 | -0.220 | -0.2768 | No |
| 196 | MSH6 | mutS homolog 6 [Source:HGNC Symbol;Acc:HGNC:7329] | 8846 | -0.221 | -0.2752 | No |
| 197 | SHH | sonic hedgehog signaling molecule [Source:HGNC Symbol;Acc:HGNC:10848] | 8941 | -0.227 | -0.2804 | No |
| 198 | MAPK8 | mitogen-activated protein kinase 8 [Source:HGNC Symbol;Acc:HGNC:6881] | 8956 | -0.228 | -0.2792 | No |
| 199 | EGLN3 | egl-9 family hypoxia inducible factor 3 [Source:HGNC Symbol;Acc:HGNC:14661] | 8965 | -0.229 | -0.2775 | No |
| 200 | PTCH2 | patched 2 [Source:HGNC Symbol;Acc:HGNC:9586] | 8994 | -0.231 | -0.2774 | No |
| 201 | SPI1 | Spi-1 proto-oncogene [Source:HGNC Symbol;Acc:HGNC:11241] | 9012 | -0.232 | -0.2764 | No |
| 202 | SMAD4 | SMAD family member 4 [Source:HGNC Symbol;Acc:HGNC:6770] | 9035 | -0.235 | -0.2758 | No |
| 203 | RUNX1 | RUNX family transcription factor 1 [Source:HGNC Symbol;Acc:HGNC:10471] | 9063 | -0.237 | -0.2755 | No |
| 204 | ABL1 | "ABL proto-oncogene 1, non-receptor tyrosine kinase [Source:HGNC Symbol;Acc:HGNC:76]" | 9081 | -0.238 | -0.2744 | No |
| 205 | ITGA6 | integrin subunit alpha 6 [Source:HGNC Symbol;Acc:HGNC:6142] | 9112 | -0.240 | -0.2744 | No |
| 206 | HSP90B1 | heat shock protein 90 beta family member 1 [Source:HGNC Symbol;Acc:HGNC:12028] | 9134 | -0.242 | -0.2736 | No |
| 207 | CSF3R | colony stimulating factor 3 receptor [Source:HGNC Symbol;Acc:HGNC:2439] | 9247 | -0.252 | -0.2799 | No |
| 208 | RET | ret proto-oncogene [Source:HGNC Symbol;Acc:HGNC:9967] | 9297 | -0.256 | -0.2813 | No |
| 209 | BAX | "BCL2 associated X, apoptosis regulator [Source:HGNC Symbol;Acc:HGNC:959]" | 9323 | -0.257 | -0.2806 | No |
| 210 | HDAC2 | histone deacetylase 2 [Source:HGNC Symbol;Acc:HGNC:4853] | 9359 | -0.260 | -0.2808 | No |
| 211 | PLD1 | phospholipase D1 [Source:HGNC Symbol;Acc:HGNC:9067] | 9365 | -0.261 | -0.2785 | No |
| 212 | ARNT | aryl hydrocarbon receptor nuclear translocator [Source:HGNC Symbol;Acc:HGNC:700] | 9427 | -0.267 | -0.2807 | No |
| 213 | CBL | Cbl proto-oncogene [Source:HGNC Symbol;Acc:HGNC:1541] | 9479 | -0.272 | -0.2820 | No |
| 214 | CBLC | Cbl proto-oncogene C [Source:HGNC Symbol;Acc:HGNC:15961] | 9553 | -0.279 | -0.2850 | No |
| 215 | FZD3 | frizzled class receptor 3 [Source:HGNC Symbol;Acc:HGNC:4041] | 9560 | -0.279 | -0.2826 | No |
| 216 | NCOA4 | nuclear receptor coactivator 4 [Source:HGNC Symbol;Acc:HGNC:7671] | 9589 | -0.282 | -0.2820 | No |
| 217 | FGF17 | fibroblast growth factor 17 [Source:HGNC Symbol;Acc:HGNC:3673] | 9693 | -0.291 | -0.2872 | No |
| 218 | MAPK10 | mitogen-activated protein kinase 10 [Source:HGNC Symbol;Acc:HGNC:6872] | 9699 | -0.291 | -0.2847 | No |
| 219 | FGF6 | fibroblast growth factor 6 [Source:HGNC Symbol;Acc:HGNC:3684] | 9739 | -0.296 | -0.2848 | No |
| 220 | ETS1 | "ETS proto-oncogene 1, transcription factor [Source:HGNC Symbol;Acc:HGNC:3488]" | 9795 | -0.301 | -0.2861 | No |
| 221 | MSH3 | mutS homolog 3 [Source:HGNC Symbol;Acc:HGNC:7326] | 9796 | -0.301 | -0.2830 | No |
| 222 | BCR | BCR activator of RhoGEF and GTPase [Source:HGNC Symbol;Acc:HGNC:1014] | 9830 | -0.304 | -0.2826 | No |
| 223 | MAP2K2 | mitogen-activated protein kinase kinase 2 [Source:HGNC Symbol;Acc:HGNC:6842] | 9932 | -0.315 | -0.2874 | No |
| 224 | FGF4 | fibroblast growth factor 4 [Source:HGNC Symbol;Acc:HGNC:3682] | 9958 | -0.317 | -0.2862 | No |
| 225 | HSP90AB1 | heat shock protein 90 alpha family class B member 1 [Source:HGNC Symbol;Acc:HGNC:5258] | 10060 | -0.327 | -0.2909 | Yes |
| 226 | TRAF5 | TNF receptor associated factor 5 [Source:HGNC Symbol;Acc:HGNC:12035] | 10085 | -0.329 | -0.2895 | Yes |
| 227 | JUP | junction plakoglobin [Source:HGNC Symbol;Acc:HGNC:6207] | 10101 | -0.331 | -0.2873 | Yes |
| 228 | SMO | "smoothened, frizzled class receptor [Source:HGNC Symbol;Acc:HGNC:11119]" | 10111 | -0.332 | -0.2847 | Yes |
| 229 | RARA | retinoic acid receptor alpha [Source:HGNC Symbol;Acc:HGNC:9864] | 10141 | -0.335 | -0.2836 | Yes |
| 230 | CTBP1 | C-terminal binding protein 1 [Source:HGNC Symbol;Acc:HGNC:2494] | 10188 | -0.339 | -0.2838 | Yes |
| 231 | MAP2K1 | mitogen-activated protein kinase kinase 1 [Source:HGNC Symbol;Acc:HGNC:6840] | 10197 | -0.340 | -0.2810 | Yes |
| 232 | EGLN2 | egl-9 family hypoxia inducible factor 2 [Source:HGNC Symbol;Acc:HGNC:14660] | 10212 | -0.342 | -0.2786 | Yes |
| 233 | ZBTB16 | zinc finger and BTB domain containing 16 [Source:HGNC Symbol;Acc:HGNC:12930] | 10246 | -0.346 | -0.2778 | Yes |
| 234 | FGF22 | fibroblast growth factor 22 [Source:HGNC Symbol;Acc:HGNC:3679] | 10261 | -0.347 | -0.2754 | Yes |
| 235 | RALGDS | ral guanine nucleotide dissociation stimulator [Source:HGNC Symbol;Acc:HGNC:9842] | 10287 | -0.349 | -0.2738 | Yes |
| 236 | FGF12 | fibroblast growth factor 12 [Source:HGNC Symbol;Acc:HGNC:3668] | 10294 | -0.350 | -0.2707 | Yes |
| 237 | CDKN2B | cyclin dependent kinase inhibitor 2B [Source:HGNC Symbol;Acc:HGNC:1788] | 10324 | -0.353 | -0.2695 | Yes |
| 238 | AXIN1 | axin 1 [Source:HGNC Symbol;Acc:HGNC:903] | 10335 | -0.354 | -0.2667 | Yes |
| 239 | FGF21 | fibroblast growth factor 21 [Source:HGNC Symbol;Acc:HGNC:3678] | 10341 | -0.355 | -0.2635 | Yes |
| 240 | APC | APC regulator of WNT signaling pathway [Source:HGNC Symbol;Acc:HGNC:583] | 10440 | -0.366 | -0.2676 | Yes |
| 241 | CASP8 | caspase 8 [Source:HGNC Symbol;Acc:HGNC:1509] | 10442 | -0.366 | -0.2639 | Yes |
| 242 | BIRC5 | baculoviral IAP repeat containing 5 [Source:HGNC Symbol;Acc:HGNC:593] | 10513 | -0.375 | -0.2657 | Yes |
| 243 | LAMC2 | laminin subunit gamma 2 [Source:HGNC Symbol;Acc:HGNC:6493] | 10531 | -0.376 | -0.2633 | Yes |
| 244 | KRAS | "KRAS proto-oncogene, GTPase [Source:HGNC Symbol;Acc:HGNC:6407]" | 10544 | -0.377 | -0.2604 | Yes |
| 245 | CCDC6 | coiled-coil domain containing 6 [Source:HGNC Symbol;Acc:HGNC:18782] | 10570 | -0.381 | -0.2585 | Yes |
| 246 | FOS | "Fos proto-oncogene, AP-1 transcription factor subunit [Source:HGNC Symbol;Acc:HGNC:3796]" | 10640 | -0.387 | -0.2601 | Yes |
| 247 | LAMC1 | laminin subunit gamma 1 [Source:HGNC Symbol;Acc:HGNC:6492] | 10642 | -0.387 | -0.2562 | Yes |
| 248 | WNT1 | Wnt family member 1 [Source:HGNC Symbol;Acc:HGNC:12774] | 10706 | -0.394 | -0.2573 | Yes |
| 249 | PLCG1 | phospholipase C gamma 1 [Source:HGNC Symbol;Acc:HGNC:9065] | 10722 | -0.395 | -0.2544 | Yes |
| 250 | CREBBP | CREB binding protein [Source:HGNC Symbol;Acc:HGNC:2348] | 10881 | -0.414 | -0.2628 | Yes |
| 251 | BMP2 | bone morphogenetic protein 2 [Source:HGNC Symbol;Acc:HGNC:1069] | 10919 | -0.420 | -0.2615 | Yes |
| 252 | PIK3R2 | phosphoinositide-3-kinase regulatory subunit 2 [Source:HGNC Symbol;Acc:HGNC:8980] | 10956 | -0.425 | -0.2600 | Yes |
| 253 | IKBKG | inhibitor of nuclear factor kappa B kinase regulatory subunit gamma [Source:HGNC Symbol;Acc:HGNC:5961] | 10986 | -0.428 | -0.2580 | Yes |
| 254 | CDK2 | cyclin dependent kinase 2 [Source:HGNC Symbol;Acc:HGNC:1771] | 11069 | -0.441 | -0.2601 | Yes |
| 255 | STK4 | serine/threonine kinase 4 [Source:HGNC Symbol;Acc:HGNC:11408] | 11074 | -0.441 | -0.2559 | Yes |
| 256 | VEGFD | vascular endothelial growth factor D [Source:HGNC Symbol;Acc:HGNC:3708] | 11095 | -0.443 | -0.2530 | Yes |
| 257 | PIK3R1 | phosphoinositide-3-kinase regulatory subunit 1 [Source:HGNC Symbol;Acc:HGNC:8979] | 11099 | -0.443 | -0.2488 | Yes |
| 258 | LAMB1 | laminin subunit beta 1 [Source:HGNC Symbol;Acc:HGNC:6486] | 11135 | -0.448 | -0.2470 | Yes |
| 259 | GSK3B | glycogen synthase kinase 3 beta [Source:HGNC Symbol;Acc:HGNC:4617] | 11157 | -0.451 | -0.2441 | Yes |
| 260 | GLI2 | GLI family zinc finger 2 [Source:HGNC Symbol;Acc:HGNC:4318] | 11195 | -0.456 | -0.2424 | Yes |
| 261 | PIAS2 | protein inhibitor of activated STAT 2 [Source:HGNC Symbol;Acc:HGNC:17311] | 11288 | -0.470 | -0.2450 | Yes |
| 262 | PPARD | peroxisome proliferator activated receptor delta [Source:HGNC Symbol;Acc:HGNC:9235] | 11301 | -0.471 | -0.2412 | Yes |
| 263 | CTNNA2 | catenin alpha 2 [Source:HGNC Symbol;Acc:HGNC:2510] | 11342 | -0.477 | -0.2395 | Yes |
| 264 | NKX3-1 | NK3 homeobox 1 [Source:HGNC Symbol;Acc:HGNC:7838] | 11348 | -0.478 | -0.2351 | Yes |
| 265 | PIAS1 | protein inhibitor of activated STAT 1 [Source:HGNC Symbol;Acc:HGNC:2752] | 11422 | -0.490 | -0.2359 | Yes |
| 266 | KITLG | KIT ligand [Source:HGNC Symbol;Acc:HGNC:6343] | 11516 | -0.503 | -0.2382 | Yes |
| 267 | WNT8B | Wnt family member 8B [Source:HGNC Symbol;Acc:HGNC:12789] | 11532 | -0.505 | -0.2343 | Yes |
| 268 | CHUK | component of inhibitor of nuclear factor kappa B kinase complex [Source:HGNC Symbol;Acc:HGNC:1974] | 11624 | -0.521 | -0.2362 | Yes |
| 269 | ITGA2B | integrin subunit alpha 2b [Source:HGNC Symbol;Acc:HGNC:6138] | 11645 | -0.524 | -0.2325 | Yes |
| 270 | EP300 | E1A binding protein p300 [Source:HGNC Symbol;Acc:HGNC:3373] | 11741 | -0.540 | -0.2346 | Yes |
| 271 | EPAS1 | endothelial PAS domain protein 1 [Source:HGNC Symbol;Acc:HGNC:3374] | 11758 | -0.542 | -0.2304 | Yes |
| 272 | HIF1A | hypoxia inducible factor 1 subunit alpha [Source:HGNC Symbol;Acc:HGNC:4910] | 11833 | -0.556 | -0.2307 | Yes |
| 273 | PTEN | phosphatase and tensin homolog [Source:HGNC Symbol;Acc:HGNC:9588] | 11836 | -0.556 | -0.2252 | Yes |
| 274 | RXRB | retinoid X receptor beta [Source:HGNC Symbol;Acc:HGNC:10478] | 11871 | -0.565 | -0.2222 | Yes |
| 275 | COL4A2 | collagen type IV alpha 2 chain [Source:HGNC Symbol;Acc:HGNC:2203] | 11940 | -0.580 | -0.2217 | Yes |
| 276 | SOS1 | SOS Ras/Rac guanine nucleotide exchange factor 1 [Source:HGNC Symbol;Acc:HGNC:11187] | 12041 | -0.603 | -0.2235 | Yes |
| 277 | MTOR | mechanistic target of rapamycin kinase [Source:HGNC Symbol;Acc:HGNC:3942] | 12047 | -0.605 | -0.2178 | Yes |
| 278 | PIAS4 | protein inhibitor of activated STAT 4 [Source:HGNC Symbol;Acc:HGNC:17002] | 12060 | -0.610 | -0.2126 | Yes |
| 279 | PDGFA | platelet derived growth factor subunit A [Source:HGNC Symbol;Acc:HGNC:8799] | 12104 | -0.621 | -0.2097 | Yes |
| 280 | EGFR | epidermal growth factor receptor [Source:HGNC Symbol;Acc:HGNC:3236] | 12109 | -0.622 | -0.2037 | Yes |
| 281 | DAPK3 | death associated protein kinase 3 [Source:HGNC Symbol;Acc:HGNC:2676] | 12180 | -0.639 | -0.2028 | Yes |
| 282 | NOS2 | nitric oxide synthase 2 [Source:HGNC Symbol;Acc:HGNC:7873] | 12262 | -0.665 | -0.2025 | Yes |
| 283 | CXCL8 | C-X-C motif chemokine ligand 8 [Source:HGNC Symbol;Acc:HGNC:6025] | 12297 | -0.676 | -0.1984 | Yes |
| 284 | WNT4 | Wnt family member 4 [Source:HGNC Symbol;Acc:HGNC:12783] | 12331 | -0.686 | -0.1941 | Yes |
| 285 | GLI1 | GLI family zinc finger 1 [Source:HGNC Symbol;Acc:HGNC:4317] | 12333 | -0.687 | -0.1872 | Yes |
| 286 | PDGFB | platelet derived growth factor subunit B [Source:HGNC Symbol;Acc:HGNC:8800] | 12389 | -0.704 | -0.1844 | Yes |
| 287 | RALA | RAS like proto-oncogene A [Source:HGNC Symbol;Acc:HGNC:9839] | 12398 | -0.707 | -0.1779 | Yes |
| 288 | LAMA3 | laminin subunit alpha 3 [Source:HGNC Symbol;Acc:HGNC:6483] | 12449 | -0.724 | -0.1746 | Yes |
| 289 | IL6 | interleukin 6 [Source:HGNC Symbol;Acc:HGNC:6018] | 12460 | -0.730 | -0.1680 | Yes |
| 290 | ITGB1 | integrin subunit beta 1 [Source:HGNC Symbol;Acc:HGNC:6153] | 12566 | -0.780 | -0.1684 | Yes |
| 291 | TCF7 | transcription factor 7 [Source:HGNC Symbol;Acc:HGNC:11639] | 12628 | -0.813 | -0.1650 | Yes |
| 292 | VEGFA | vascular endothelial growth factor A [Source:HGNC Symbol;Acc:HGNC:12680] | 12629 | -0.813 | -0.1568 | Yes |
| 293 | PTGS2 | prostaglandin-endoperoxide synthase 2 [Source:HGNC Symbol;Acc:HGNC:9605] | 12641 | -0.820 | -0.1494 | Yes |
| 294 | CDC42 | cell division cycle 42 [Source:HGNC Symbol;Acc:HGNC:1736] | 12681 | -0.852 | -0.1439 | Yes |
| 295 | IGF1R | insulin like growth factor 1 receptor [Source:HGNC Symbol;Acc:HGNC:5465] | 12689 | -0.855 | -0.1358 | Yes |
| 296 | LAMA5 | laminin subunit alpha 5 [Source:HGNC Symbol;Acc:HGNC:6485] | 12692 | -0.859 | -0.1272 | Yes |
| 297 | COL4A1 | collagen type IV alpha 1 chain [Source:HGNC Symbol;Acc:HGNC:2202] | 12703 | -0.866 | -0.1192 | Yes |
| 298 | TRAF4 | TNF receptor associated factor 4 [Source:HGNC Symbol;Acc:HGNC:12034] | 12706 | -0.867 | -0.1106 | Yes |
| 299 | CBLB | Cbl proto-oncogene B [Source:HGNC Symbol;Acc:HGNC:1542] | 12725 | -0.887 | -0.1031 | Yes |
| 300 | CDKN1A | cyclin dependent kinase inhibitor 1A [Source:HGNC Symbol;Acc:HGNC:1784] | 12727 | -0.890 | -0.0942 | Yes |
| 301 | HSP90AA1 | heat shock protein 90 alpha family class A member 1 [Source:HGNC Symbol;Acc:HGNC:5253] | 12730 | -0.893 | -0.0853 | Yes |
| 302 | NFKB2 | nuclear factor kappa B subunit 2 [Source:HGNC Symbol;Acc:HGNC:7795] | 12750 | -0.912 | -0.0776 | Yes |
| 303 | MYC | "MYC proto-oncogene, bHLH transcription factor [Source:HGNC Symbol;Acc:HGNC:7553]" | 12801 | -0.970 | -0.0717 | Yes |
| 304 | JUN | "Jun proto-oncogene, AP-1 transcription factor subunit [Source:HGNC Symbol;Acc:HGNC:6204]" | 12813 | -0.994 | -0.0625 | Yes |
| 305 | NFKBIA | NFKB inhibitor alpha [Source:HGNC Symbol;Acc:HGNC:7797] | 12817 | -1.001 | -0.0526 | Yes |
| 306 | FN1 | fibronectin 1 [Source:HGNC Symbol;Acc:HGNC:3778] | 12820 | -1.005 | -0.0426 | Yes |
| 307 | PGF | placental growth factor [Source:HGNC Symbol;Acc:HGNC:8893] | 12850 | -1.059 | -0.0342 | Yes |
| 308 | CTNNB1 | catenin beta 1 [Source:HGNC Symbol;Acc:HGNC:2514] | 12866 | -1.119 | -0.0241 | Yes |
| 309 | E2F3 | E2F transcription factor 3 [Source:HGNC Symbol;Acc:HGNC:3115] | 12886 | -1.224 | -0.0132 | Yes |
| 310 | AKT2 | AKT serine/threonine kinase 2 [Source:HGNC Symbol;Acc:HGNC:392] | 12896 | -1.404 | 0.0003 | Yes |
Table: GSEA details [plain text format]

  

Fig 2: KEGG\_PATHWAYS\_IN\_CANCER      
 Blue-Pink O' Gram in the Space of the Analyzed GeneSet

  

Fig 3: KEGG\_PATHWAYS\_IN\_CANCER: Random ES distribution      
 Gene set null distribution of ES for **KEGG\_PATHWAYS\_IN\_CANCER**
